# Changes in cerebrospinal fluid and serum cytokine levels in severe traumatic brain injury patients

**DOI:** 10.1186/cc10896

**Published:** 2012-03-20

**Authors:** T Saito, H Kushi, J Sato, A Yoshino, K Tanjo

**Affiliations:** 1 Nihon University, School of Medicine, Tokyo, Japan; 2 Nihon University, College of Humanities and Sciences, Tokyo, Japan

## Introduction

Inflammatory response following brain injury begins with brain tissue injury triggered neuroinflammation, which induces a systemic inflammatory response syndrome. We investigated the characteristics of the acute inflammatory response following severe traumatic brain injury through changes in cerebrospinal fluid (CSF) and serum cytokine levels.

## Methods

The subjects were 24 patients with severe traumatic brain injury. We measured levels of the proinflammatory cytokines IL-6 and IL-8, and the anti-inflammatory cytokine IL-10 in peripheral blood and CSF on four occasions, at the time of admission and after 24 hours, 72 hours and 1 week.

## Results

CSF and serum IL-6 levels continued to rise until 72 hours after admission. CSF IL-6 levels were 50 to 400 times serum levels. Serum IL-8 levels remained at 20 to 30 pg/ml. CSF IL-8 levels were 100 to 800 times the serum levels, and remained high after the peak of 23,500 pg/ml at the time of admission. CSF and serum IL-10 levels were high, but not abnormally high as for IL-6 and IL-8, and decreased with time. The difference in CSF and serum levels, as seen for IL-6 and IL-8, was not seen for IL-10.

## Conclusion

We elucidated the following points concerning the acute inflammatory response following severe traumatic brain injury. High levels of IL-6 and IL-8 are maintained in both CSF and serum. CSF levels of IL-6 and IL-8 are one or two orders of magnitude greater than serum levels. Upregulation of IL-10 is minimal in comparison with IL-6 and IL-8, suggesting that in neuroinflammation IL-10 functions poorly as an anti-inflammatory cytokine.

